# Commentary: Tissue factor as a potential coagulative/vascular marker in relapsing-remitting multiple sclerosis

**DOI:** 10.3389/fimmu.2023.1320179

**Published:** 2023-12-01

**Authors:** Nigel Mackman, Ana T.A. Sachetto

**Affiliations:** Department of Medicine, University of North Carolina at Chapel Hill, Chapel Hill, NC, United States

**Keywords:** multiple sclerosis, tissue factor, inflammation, biomarker, coagulation

Koudriavtseva and colleagues (1) measured levels of different biomarkers in healthy controls, patients with remitting multiple sclerosis (MS), and patients with relapsing MS. The Quantikine enzyme-linked immunosorbent assay (ELISA) (R&D Systems, Minneapolis, MN, Cat. No. DCF300) was used to measure plasma tissue factor (TF) (S. Lorenzano, personal communication). The authors found that patients with relapsing MS had lower levels of plasma TF compared to either patients with remitting MS or the control group. It was concluded that plasma TF is a promising biomarker and possible therapeutic target in relapsing-remitting MS.

A previous clinical study called Comparison of Acute Treatments in Cancer Hemostasis (CATCH) used the same Quantikine ELISA to measure plasma TF levels in 805 patients with cancer and acute symptomatic venous thromboembolism treated with anticoagulants (2). The mean and median values for TF were 72.5 pg/mL and 50.3 pg/mL, respectively. Plasma TF was identified as a biomarker of recurrent venous thromboembolism in this group of patients. We were invited to write a commentary on the paper (understanding the pathway), in which we raised concerns about the ability of the Quantikine ELISA to accurately measure levels of TF in human plasma (3).

Recently, we assessed the ability of four commercial human TF ELISAs, including the Quantikine ELISA, to detect TF in various biological samples, including human plasma (4). Two of the ELISAs (Quantikine and Imubind) detected recombinant TF and TF in extracellular vesicles from a TF-expressing human pancreatic cell line. However, the ELISAs failed to detect TF in plasma that contained TF-positive extracellular vesicles (1.2–2.6 pg/mL) (determined using a factor Xa generation assay). The only plasma samples that had a signal above the background of plasma from healthy controls were from three patients with acute leukemia, who had very high levels of extracellular vesicle TF activity (151–696 pg/mL). We concluded that, except for some patients with leukemia, the Quantikine ELISA could not detect TF in human plasma.

There are two significant challenges with measuring TF in human plasma. First, the concentration of this potent procoagulant protein is very low. Secondly, plasma contains multivalent substances, such as heterotypic antibodies, that can generate signals in ELISAs independently of the target protein by simply bridging assay antibodies. Such heterotypic antibodies may increase in different disease states. The datasheet for the Quantikine ELISA states that the mean value for plasma from healthy controls is 33.3 pg/mL (**Table 1**). We and others (4–7) have observed similar low values for healthy individuals (mean, 23.5–45.1 pg/mL; median, 39.1–52.4 pg/mL) (**Table 1**). In contrast, Koudriavtseva and colleagues (1) report much higher values of TF in healthy controls (mean, 110.85 pg/mL; median, 120.69 pg/mL) (**Table 1**). Patients with remitting MS had similarly high values (mean, 94.06 pg/mL; median, 99.23 pg/mL), whereas patients with relapsing MS had low values (mean, 45.03 pg/mL; median, 22.72 pg/mL). These low values are more consistent with healthy controls than a disease group.

**Table 1:** Measurement of the levels of plasma tissue factor in healthy controls and patients with multiple sclerosis using the quantikine TF ELISA.

	Mean (SD) (pg/mL)	Median (IQR) (pg/mL)	Ref.
Healthy control (*n* = 36)	33.3 (6.6)		R&D datasheet
Healthy control (*n* = 20)	–	52.4 (28.4–72.4)	(5)
Healthy control (*n* = 41)	–	39.1 (16.8–87.3)	(6)
Healthy control (*n* = 40)	23.5 (5)	–	(7)
Healthy control (*n* = 5)	45.1 (14.7)	40.6 (33.4–59.0)	(4)
Healthy control (*n* = 30)	110.85 (56.31)	120.69^a^	(1)
Remitting MS (*n* = 30)	94.06 (55.83)	99.23^a^	(1)
Relapsing MS (*n* = 30)	45.03 (37.66)	22.72^a^	(1)

SD, standard deviation; IQR, interquartile range.

a A single value was reported for the IQR instead of a range.

We found that the Quantikine ELISA could not detect low levels of TF in human plasma. We believe that additional methods should be used to demonstrate changes in TF levels in the plasma, such as measuring extracellular vesicle TF activity, before concluding that plasma TF is a biomarker of relapsing-remitting MS.

## Author contributions

NM: Writing – original draft. AS: Writing – review & editing.

## References

[1] Koudriavtseva T Lorenzano S Cellerino M Truglio M Fiorelli M Lapucci C. Tissue factor as a potential coagulative/vascular marker in relapsing-remitting multiple sclerosis. Front Immunol (2023) 14:1226616. doi: 10.3389/fimmu.2023.1226616 37583699 PMC10424925

[2] Khorana AA Kamphuisen PW Meyer G Bauersachs R Janas MS Jarner MF. Tissue factor as a predictor of recurrent venous thromboembolism in Malignancy: biomarker analyses of the CATCH trial. J Clin Oncol (2017) 35(10):1078–85. doi: 10.1200/JCO.2016.67.4564 28029329

[3] Ay C Mackman N. Tissue factor: catch me if you can! J Clin Oncol (2017) 35(10):1128–30. doi: 10.1200/JCO.2016.70.6788 28029321

[4] Sachetto ATA Archibald SJ Bhatia R Monroe D Hisada Y Mackman N. Evaluation of four commercial ELISAs to measure tissue factor in human plasma. Res Pract Thromb Haemostasis (2023) 100133. doi: 10.1016/j.rpth.2023.100133 PMC10233285 37275179

[5] Talar-Wojnarowska R Woźniak M Borkowska A Cypryk K Olakowski M Małecka-Panas E. Procoagulant disorders in patients with newly diagnosed pancreatic adenocarcinoma. Medicina (2020) 56(12):677. doi: 10.3390/medicina56120677 33316933 PMC7763230

[6] Kobayashi S Koizume S Takahashi T Ueno M Oishi R Nagashima S. Tissue factor and its procoagulant activity on cancer-associated thromboembolism in pancreatic cancer. Cancer Sci (2021) 112(11):4679–91. doi: 10.1111/cas.15106 PMC8586686 34382298

[7] Cacciola R Gentilini Cacciola E Vecchio V Cacciola E. Cellular and molecular mechanisms in COVID-19 coagulopathy: role of inflammation and endotheliopathy. J Thromb Thrombolysis (2022) 53(2):282–90. doi: 10.1007/s11239-021-02583-4 PMC8536904 34687400

